# Characteristics and Bioactivities of Protein Hydrolysate from Cricket (*Acheta domesticus*) Powder Defatted Using Ethanol with Aid of Vacuum Impregnation

**DOI:** 10.3390/foods13203250

**Published:** 2024-10-13

**Authors:** Lalita Chotphruethipong, Theeraphol Senphan, Avtar Sigh, Pilaiwanwadee Hutamekalin, Pornpot Nuthong, Soottawat Benjakul

**Affiliations:** 1 Department of Food Science, Faculty of Science, Burapha University, Mueang Chonburi, Chonburi 20131, Thailand; 2 Program in Food Science and Technology, Faculty of Engineering and Agro-Industry, Maejo University, Sansai, Chiangmai 50290, Thailand; theeraphol_s@mju.ac.th; 3 International Center of Excellence in Seafood Science and Innovation, Faculty of Agro-Industry, Prince of Songkla University, Hat Yai, Songkhla 90110, Thailand; avtar.s@psu.ac.th (A.S.); soottawat.b@psu.ac.th (S.B.); 4 Division of Health and Applied Sciences, Faculty of Science, Prince of Songkla University, Hat Yai, Songkhla 90110, Thailand; pilaiwanwadee.h@psu.ac.th; 5 Office of Scientific Instrument and Testing, Prince of Songkla University, Hat Yai, Songkhla 90110, Thailand; pornpot.n@psu.ac.th

**Keywords:** defatting process, vacuum impregnation, cricket, protein hydrolysate, characterization, bioavailability

## Abstract

Cricket is a potential proteinaceous source used for protein hydrolysate (PH) preparation, having several biological activities. Nevertheless, cricket has high lipid contents, which are susceptible to oxidation during PH preparation. Thus, ethanol was used together with vacuum impregnation (VI) to enhance defatting efficacy before PH preparation. Also, bioavailability of the digest of PH after gastrointestinal tract (GIT) digestion via the Caco-2 monolayer was assessed. Cricket powder was defatted using ethanol for 1–4 h. Lipid contents were decreased with enhancing time until 2 h. Additionally, the defatting efficacy was augmented when ethanol combined with VI at 4 cycles for 2 h (VI-E-2) was implemented. Lowered mono- and polyunsaturated fatty acid contents were also observed in the VI-E-2 sample. The VI-E-2 sample was used to prepare PH using Alcalase and Flavourzyme (0.2–0.4 units/g dry sample). PH prepared by Alcalase hydrolysis at 0.2 units/g dry sample (A-0.2) showed the higher ABTS radical-scavenging activity and FRAP, compared to that prepared by Flavourzyme hydrolysis (*p* < 0.05). Thus, the A-0.2 sample was selected for digestion via the GIT system. The obtained digest (500–1000 μg/mL) had bioavailability of peptides, depending on the levels used. Therefore, PH from defatted cricket powder could be a promising ingredient for food applications.

## 1. Introduction

Nowadays, consumers focus on health promotion. As a consequence, functional ingredients or nutraceuticals have gained remarkable interest, particularly from edible insects, considered as future food. Cricket is considered an attractive feedstock for manufacturing functional ingredients since it contains numerous nutrients, including protein (60–70%) and lipids (10–23%) [1]. The protein isolate and concentrate from cricket can facilitate their use as functional ingredients of insects in the food industry, especially human diets. Moreover, proteins obtained from edible insects have gained increasing demand for both domestic consumption and export. Also, they have less greenhouse gas and ammonia emissions, making them more environmentally friendly [2]. They constitute a new and accessible business opportunity for developing countries as they are relatively fast, easy, and inexpensive to farm, which makes them the overall best option to ensure food security with the ever-growing worldwide population [3]. Also, insect proteins have many favorable attributes such as high nutritional value and digestibility [4].

House cricket (*Acheta domesticus*), Mang Sa Ding, is significant for the Thai economy because of its well-developed farming system. Cricket has been cultured for more than 20,000 smallholder farmers in Thailand [5], especially in Northeastern, Northern, and Eastern Thailand. Several studies reported that protein isolated from cricket has excellent functional properties [5,6]. Also, the protein hydrolysate (PH) from cricket possesses versatile bioactivities such as antioxidant and antihypertensive activities [5,7,8]. Different hydrolysis processes and the types of proteases as well as enzyme concentrations are essential factors affecting the bioactivity and size of peptides. Alcalase and Flavourzyme are commonly applied for hydrolyzing the protein of crickets [7,9]. PH from cricket (*Gryllus bimaculatus*) prepared using Alcalase showed high ABTS radical-scavenging activity (ABTS-RSA) (0.44–0.55 µmol TE eq./g) and metal chelating activity (MCA) (1721.99–1751.71 µmol EE eq./g) [8]. PH from black cricket (*Gryllus assimilis*) prepared by Flavourzyme^TM^, 500 L, showed powerful antioxidant properties, including DPPH-RSA (IC_50_ = 455 µg/mL) and ABTS-RSA (IC_50_ = 71 µg/mL) [10]. Even though cricket PH has several bioactive peptides, it contains high amounts of fat or lipids, constituting mono- and polyunsaturated fatty acids [11], which can be oxidized during hydrolysis at high temperatures. This phenomenon led to the development of undesirable compounds, especially rancid odorous components in the final products that might be a critical problem for further supplements in foods. To overcome this obstacle, defatting has been implemented. Hexane has been generally used for the defatting of edible insects [11,12]. Although this technique could reduce the fat content in insects effectively, environmental, economic, and safety concerns are disadvantages. Ethanol is an alternative solvent for the defatting of insects, in which lipids from insects can be removed to a high degree [13]. To enhance the defatting efficacy by ethanol, the vacuum impregnation (VI) process could be the potential method according to the hydrodynamic mechanism [14], leading to the increased permeation of the solvent into the pores of cricket powders. Thus, lipids localized in cricket could be extracted to a higher extent. The ethanol with the aid of the VI process might augment the removal of lipids from cricket before PH preparation. This technique could be a potential means to reduce unwanted odor occurring in extracted proteins and their products [15]. Consequently, PH from defatted cricket could be applied as a food ingredient for the production of functional foods, especially as a protein source for food fortification with high bioactivities and safety. Since no information on the defatting process using VI along with ethanol for cricket powders before hydrolysate preparation exists, this study aimed (1) to elucidate the impact of VI along with ethanol for the defatting of cricket powders and (2) to investigate the influence of two different enzymatic hydrolyses on characteristics, antioxidative activities, and bioavailability of the resulting protein hydrolysates. The information gained could be beneficial for the production of natural ingredients, particularly for superfood, with high quality. In addition, edible insect resources could be better exploited, which is related to sustainable development goals (SDGs) in terms of healthy lives and the promotion of well-being.

## 2. Materials and Methods

### 2.1. Enzymes and Chemicals

Alcalase was given by Siam Victory Chemicals Co, Ltd. (Bangkok, Thailand). Flavourzyme^®^ was provided by Novozymes (Basvaerd, Denmark). All chemicals were of an analytical grade. Chemicals for measuring antioxidant activities, including 2,2-diphenyl-1-picrylhydrazyl (DPPH), 2,2′-azino-bis (3-ethylbenzthiazoline-6-sulphonic acid (ABTS), 2,4,6-Tri(2-pyridyl)-s-triazine (TPTZ), and Ethylenediaminetetraacetic acid (EDTA), were supplied by Sigma-Aldrich Chemical Co (St. Louis, MO, USA). Fetal bovine serum (FBS) and antibiotics (penicillin and streptomycin) were procured by Gibco BRL Life Technologies (Grand Island, NY, USA). A human colon adenocarcinoma cell line (Caco-2 cells) was provided by ATCC (Wesel, North Rhine-Westphalia, Germany).

### 2.2. Preparation of Cricket Powder

**Figure 1:**
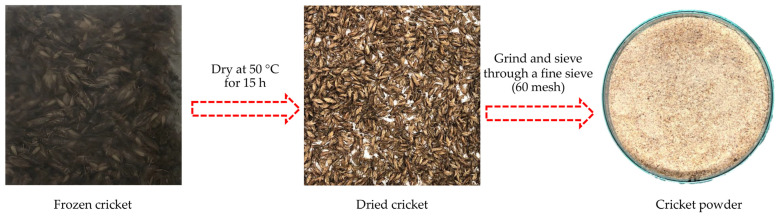
Preparation of cricket powder.

Frozen cricket was obtained from a cricket farm (Ubon-Ratchatani, Thailand). Samples were cleaned and dried using a tray dryer at 50 °C for 15 h, until the remaining moisture content of samples reached a 6.02 ± 0.04%wet basis. Thereafter, the dried samples were ground and sieved through a fine sieve (60 mesh) (Figure 1). The resulting powder was then kept at −20 °C until use.

### 2.3. Defatting of Cricket Powder Using Ethanol at Different Times

For the conventional method, the cricket powder was mixed with 5 volumes of a 95% ethanol solution, and stirred (150 rpm, room temperature) with an overhead stirrer equipped with a propeller (RW 20.n, IKA Labortechnik, Staufen, Germany) for different times (1, 2, 3, and 4 h) at 25 ± 5 °C. The solvent was removed by centrifugation at 12,298× *g* for 20 min at 25 °C [15]. The sediment was collected and dried on an aluminum foil tray in a fume hood until the solvent was completely removed. Remaining lipids in the samples were extracted using a Soxhlet extractor (Soxtherm Gerhardt Variostat, Wiesbaden, Germany) with petroleum ether as a solvent [16]. The dried samples (5 g) were loaded in a thimble filter and extracted with 150 mL of petroleum ether. The extraction proceeded at 140 °C for 3 h. After oil extraction, mixtures were kept in a round-bottom flask and the solvent was then evaporated. The resulting oils were dried in an oven at 105 °C until a constant weight was gained. Extraction time rendering the highest lipid removal was selected for the next study.

### 2.4. Defatting of Cricket Powder Using Ethanol with the Aid of Vacuum Impregnation (VI)

For VI in combination with the ethanol process, the VI system contained a 3 L vacuum chamber equipped with a vacuum pump (VE115N, Zhejiang Value Mechanical & Electrical Products Co., Ltd., China). Before the VI process, the powder was placed in a vacuum chamber containing a 95% ethanol solution with the powder/solvent ratio of 1:5 (w/v) for 5 min. The VI process was subsequently conducted at various VI cycles (1, 2, 3, and 4) with a pressure of 10 ± 5 kPa, in which one cycle of VI took 15 min for vacuum time and 10 min for restoration to atmospheric pressure with a total operation time of 2 h. After the defatting process, the samples were centrifuged, and the sediment was collected. All samples were measured for lipid content using the Soxhlet extraction method [16]. The condition of the VI cycle with the highest defatting efficacy was chosen for pre-treatment of the sample before the protein hydrolysate (PH) preparation. The powder defatted using ethanol with the aid of VI was named as ‘VI-E’. All samples, including E, VI-E, and non-defatted cricket powder (control), were analyzed as follows:

#### 2.4.1. Fatty Acid Profile of Extracted Residual Fat Using GC-FID

Fatty acid methyl esters (FAMEs) were prepared as detailed by Ali et al. [17]. The FAMEs (1 μL) were subjected to a gas chromatography (GC) system, 7890B Series (Agilent Technologies, Santa Clara, CA, USA), equipped with a flame ionization detector (FID) and a CP-Sil 88 capillary. The identified peaks were integrated and calibrated against the standard curve using Open LAB CDS software (Chem Station edition, Agilent Technologies, Santa Clara, CA, USA).

#### 2.4.2. Lipid Distribution by Confocal Laser Scanning Microscope (CLSM)

The samples, including the control, E, and VI-E, were suspended in a 1% Nile blue A solution for 10 min. Thereafter, lipids in the samples were visualized by the CLSM in the fluorescence mode (LSM 800, Zeiss, Germany), where the excitation and the emission wavelengths were at 533 nm and 630 nm, respectively [18].

### 2.5. Preparation of Defatted Cricket Protein Hydrolysate (DC-PH)

#### 2.5.1. Determination of Enzyme Activities

Alcalase and Flavourzyme were tested for their activities using casein as a substrate. The enzyme activity was assessed at pH 8.0 and 50 °C for 15 min for Alcalase and at pH 7.0 and 55 °C for 15 min for Flavourzyme. The resulting peptide content was measured using the Lowry assay [19], where tyrosine was used as a standard. One unit of activity was defined as the amount of Alcalase or Flavourzyme that liberated 0.01 μmol of tyrosine per min (μmol Try/min) [20].

#### 2.5.2. DC-PH Preparation

The selected sample rendering the lowest lipid content (obtained from Section 2.4) was prepared for PH. Briefly, dried cricket powder (50 g) was mixed with 500 mL of distilled water, followed by pH adjustment at pH 8 for Alcalase hydrolysis or pH 7 for Flavourzyme hydrolysis. Before hydrolysis, the samples were pre-incubated in a water bath at the optimum temperature for each enzyme tested. Thereafter, proteases at three different concentrations (0.2, 0.3, and 0.4 units/g dry sample) were added to the samples, in which hydrolysis temperatures and times used for Alcalase and Flavourzyme were 50 °C for 120 min and 55 °C for 90 min, respectively. Proteolytic hydrolysis conditions were obtained from a preliminary study, which showed the highest degree of hydrolysis. After hydrolysis, the reaction was terminated at 90 °C for 15 min, followed by centrifugation (9000× *g*, 20 min). The supernatant was subjected to lyophilization [21]. All the samples were measured for α-amino group content as explained by Theerawitayaart et al. [22]. The result was presented as mmol L-leucine/g dry sample.

The antioxidant activities, including scavenging activity towards DPPH^•^ and ABTS^•+^, ferric reducing activity power (FRAP), and MCA, were investigated [20,21]. The results for activity towards DPPH^•^ and ABTS^•+^ and FRAP were expressed as µmol Trolox (TE)/g dry sample. For MCA, the activity was reported as µmol EDTA/g dry sample. Ascorbic acid at 0.1 mg/mL was used as the positive control for comparing the antioxidant activities to protein hydrolysates.

The sample providing the best results based on both α-amino group content and antioxidant activities was selected for further characterization.

#### 2.5.3. Characterization of DC-PH

The selected DC-PH was analyzed as follows:

##### Size Distribution of Peptides in DC-PH

A matrix-assisted laser desorption/ionization time-of-flight (MALDI-TOF) mass spectrometer equipped with a 337 nm and N_2_ laser was used to analyze the size of peptides (Bruker Autoflex Speed, Bruker Daltonik, GmbH, Breman, Germany) [23].

##### Amino Acid Composition

The selected sample was analyzed for amino acids using an amino acid analyzer (MLC-703; Atto Co., Tokyo, Japan) [20]. The amount of amino acid was expressed as residues/1000 residues.

#### 2.5.4. Bioavailability of the Digests Obtained from Gastrointestinal Tract (GIT) Digestion across Caco-2 Monolayer

##### Cell Culture

Caco-2 cells were cultured in Eagle’s minimum essential medium (Corning, Inc., Corning, NY, USA), having 10% FBS, 1% L-glutamine, and 100 µg/mL of antibiotics. Thereafter, cells were incubated in an incubator at 37 °C with 95% air, and 5% CO_2_ [23].

##### Cell Viability

The 3-(4,5-dimethylthiazol-2-yl)-2,5-diphenyltetrazolium bromide (MTT) (Sigma-Aldrich) assay was used to assess the cell viability of the selected DC-PH after GIT digestion in Caco-2 cells [23]. Cells (1 × 10^4^ cells/well) were initially treated with the digests obtained from GIT at different concentrations (100, 250, 500, 750, and 1000 µg/mL). Cells were subsequently incubated in an incubator for 24 h and viability was determined using the MTT assay. The concentrations without cytotoxicity were chosen for the bioavailability study.

##### Bioavailability Study

Caco-2 cells (1 × 10^5^ cells/cm^2^) were cultured on a transwell plate (12-well plate, 24 mm diameter, 0.4 µm pore size membrane, Costar Corp., Corning, NY, USA) for 21 days to fully differentiate the cells [23]. The medium was changed every 2 days. The integrity of cell monolayers was monitored using a transepithelial electrical resistance (TEER) voltohmmeter (Millicell-ERS, Millipore, Ireland), in which TEER values were >300 Ω·cm^2^. For the bioavailability study, serum-free media (1.5 mL) and the digests (50 µL) at the selected levels were added to the apical side of the 12 mm transwell^®^ plate (Corning, Inc.). Serum-free media (2 mL) were added to the basolateral side. The cells were incubated for 2 h in a cell incubator. The basolateral medium was then collected and measured for the α-amino group content (AAGC) [22]. The bioavailability of peptides was calculated by the following equation:

$$\mathrm{Bioavailability}\;\left(\%\right)=\frac{{\mathrm{AAGC}}_{\mathrm{basolateral}}}{{\mathrm{AAGC}}_{\mathrm{added}\;\mathrm{digest}}}\times 100$$

### 2.6. Statistical Analysis

A Completely Randomized Design was used for all trials. All data were presented as the average ± standard deviation (SD). An analysis of variance (ANOVA) was conducted and mean comparison was performed by Duncan’s multiple range test [24].

## 3. Results

### 3.1. Impact of Ethanol at Various Extraction Times on Defatting of Cricket Powder

The defatting efficacy of cricket powder as influenced by ethanol at various extraction times is presented in Table 1. The effectiveness of ethanol for the defatting of cricket powder enhanced with increasing times (*p* < 0.05). Extraction times longer than 2 h did not increase the defatting efficacy (*p* > 0.05). Thus, the appropriate extraction time using ethanol for defatting was 2 h, in which lipids were removed by 80.38% from cricket powder.

### 3.2. Impact of Ethanol in Combination with Vacuum Impregnation (VI) in Different VI Cycles on Defatting of Cricket Powder

Table 2 shows the effectiveness of ethanol in combination with VI in different vacuum cycles (1, 2, 3, and 4) on the lipid removal of cricket powder. Overall, the augmented VI cycles led to the increase in lipid removal of cricket powder (*p* < 0.05). The highest efficacy of lipid removal of cricket powder was observed by using ethanol along with VI for four cycles (*p* < 0.05), where lipids were removed by 86.83%. Moreover, the use of the VI process increased the defatting of cricket powder more effectively than using ethanol alone (E-2) (*p* < 0.05). The finding demonstrated that the defatting of cricket powder using ethanol with the aid of VI was more effective than the solvent extraction alone as witnessed by higher lipid reduction (*p* < 0.05), compared to that defatted with the E-2 process (Table 2). Since the use of ethanol along with VI in four cycles showed the highest efficacy for lipid removal, this process was thus selected for the next study.

### 3.3. Fatty Acid Profiles of Remaining Lipids in Cricket Powder as Influenced by Different Selected Defatting Processes

**Figure 2:**
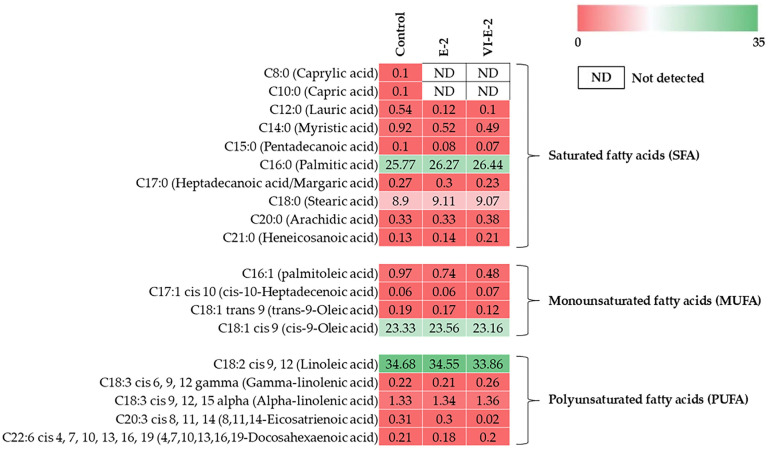
A heat map of fatty acid profiles in cricket powder as affected by different defatting processes. The color gradient is the amount of fatty acids (g/100 g lipid). Control: non-defatted cricket powder; E-2: cricket powder defatted with ethanol for 2 h; VI-E-2: cricket powder defatted with ethanol with the aid of vacuum impregnation (VI) with a total operation time of 2 h.

Fatty acid profiles of the remaining lipids in the control (without defatting processes), E-2, and VI-E-2 samples are presented in Figure 2. Lipids extracted from the control had 37.17% saturated fatty acids (SFAs), 24.55% monounsaturated fatty acids (MUFAs), and 36.75% polyunsaturated fatty acids (PUFAs), while SFA, MUFA, and PUFA contents of lipids extracted from the VI-E-2 sample were 36.98%, 23.84%, and 35.70%, respectively. Linoleic acid was found as the major fatty acid. Oleic acid constituted a high content, followed by palmitic acid in all the samples. Cricket powder defatted with E-2 and VI-E-2 processes had the lower PUFA contents (35.70%–36.57%), compared to the control sample (36.75%), especially linoleic acid, 8,11,14-eicosatrienoic acid, and 4,7,10,13,16,19-docosahexaenoic acid (DHA). When the lipid contents between E-2 and VI-E-2 samples were compared, the latter had lower MUFA and PUFA contents than the former. The lowered residual MUFA and PUFA contents in VI-E-2 samples suggested that VI could be used as a promising method for increasing the efficacy of the defatting of cricket powder.

### 3.4. Lipid Distribution in Cricket Powder as Influenced by Different Defatting Processes

**Figure 3:**
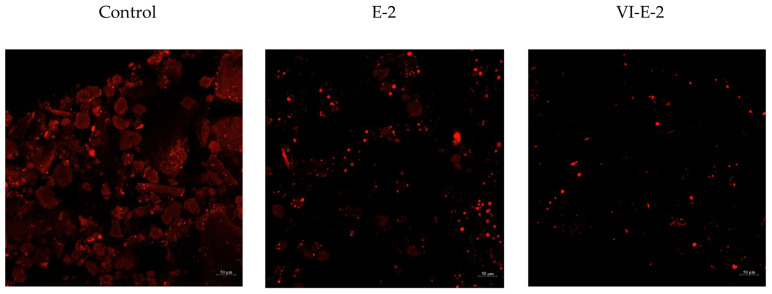
CLSM images of lipid distribution in cricket powder as affected by different selected defatting processes. Control: non-defatted cricket powder; E-2: cricket powder defatted with ethanol for 2 h; VI-E-2: cricket powder defatted with ethanol with the aid of vacuum impregnation (VI) with a total operation time of 2 h.

Lipid distribution in cricket powder as affected by different defatting processes is illustrated in Figure 3. The control (non-defatted powder) had the highest lipid distribution, as compared to other samples. This was related to the higher MUFA and PUFA contents (Figure 2). When considering lipid distribution in cricket powder using different defatting processes, the sample subjected to the defatting process using ethanol alone (E-2) showed the higher lipid distribution than that using vacuum impregnation combined with ethanol (VI-E-2). The result was in line with the lipid contents (Table 2), supporting that the use of VI was the effective means for lipid removal in cricket powder.

### 3.5. Effect of Different Enzymatic Hydrolysis on Yield, **α**-Amino Group Content, and Antioxidative Activities of Protein Hydrolysate from Defatted Cricket Powder (DC-PH)

#### 3.5.1. % Yield

Protein hydrolysate from defatted cricket powder (DC-PH) prepared using different enzymatic hydrolysis processes affected yield, α-amino group content, and antioxidative activities differently (Table 3). DC-PH prepared using Alcalase (A-0.2, A-0.3, and A-0.4) showed a higher yield than that digested by Flavourzyme (F-0.2, F-0.3, and F-0.4) at all the concentrations tested (*p* < 0.05). It was noted that the use of Alcalase hydrolysis favored the cleavage of cricket protein compared to the use of Flavourzyme hydrolysis (*p* < 0.05). When considering the concentrations tested within the same enzyme used, the highest yield was found with increasing concentrations of Alcalase and Flavourzyme until 0.3 units/g dry sample (*p* < 0.05), suggesting that the yield of the resulting DC-PH was governed by the level of enzyme used.

#### 3.5.2. The α-Amino Group Content

The α-amino group contents in DC-PH samples ranged from 4.73 to 6.27 mmol L-leucine/g dry sample (Table 3). DC-PHs prepared by Alcalase hydrolysis had higher α-amino group content than that prepared by Flavourzyme hydrolysis at the same concentration used (*p* < 0.05). It was noted that hydrolysis with Alcalase augmented the breakdown of peptide bond in defatted cricket protein more potently than hydrolysis with Flavourzyme. Moreover, the increase in enzyme concentration did not increase cleavage of peptides in DC-PHs (*p* > 0.05).

#### 3.5.3. Antioxidant Activities

Antioxidant activities of DC-PH as influenced by protease types and enzyme concentrations are shown in Table 3. Overall, DC-PHs could quench radicals and chelate metal ions. Also, it could be a reducing agent. The DC-PH prepared by Alcalase hydrolysis exhibited higher antioxidant activities than that prepared with Flavourzyme at all concentrations tested (*p* < 0.05). This finding reflected that Alcalase hydrolysis could produce peptides with antioxidant activities more effectively than Flavourzyme. The highest ABTS radical-scavenging activity, FRAP, and MCA were observed in the A-0.2 sample, compared to other samples (*p* < 0.05). When considering the concentration of the enzyme tested, the increased concentration of both enzymes did not increase antioxidant activities in all assays tested (*p* < 0.05), suggesting that a certain enzyme concentration might not increase the release of antioxidative peptides. When the samples tested were compared to ascorbic acid, the antioxidant activities of the samples tested were lower than ascorbic acid in all assays tested (*p* < 0.05), except MCA, in which the A-0.2 and F-0.2 samples had activity higher than ascorbic acid (*p* < 0.05). Moreover, there was no difference in MCA between the samples (A-0.3 and A-0.4) and ascorbic acid (*p* > 0.05). Since the A-0.2 sample rendered the highest α-amino group content and antioxidant activities, it was selected for further studies.

### 3.6. The Size Distribution of the Defatted Cricket Protein Hydrolysate Prepared Using Alcalase at 0.2 Units/g Dry Sample (A-0.2 Sample)

**Figure 4:**
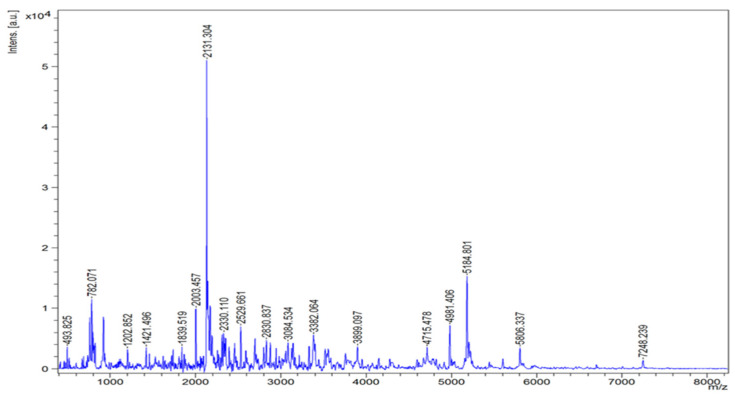
The size distribution of the defatted cricket protein hydrolysate prepared using Alcalase at 0.2 units/g dry sample (A-0.2 sample) assessed by MALDI-TOF mass spectrometry.

Peptides having varied molecular weights (MWs) were found in the selected DC-PH (A-0.2 sample) (Figure 4), where peptides with MWs of 2131 and 5184 Da were dominant in DC-PH. Moreover, peptides with low MW (<1 kDa) and large MW (>5 kDa) were also observed.

### 3.7. Amino Acid Composition of A-0.2 Sample

The amino acid composition of the A-0.2 sample is presented in Table 4. The A-0.2 sample contained essential amino acids (387.07 residues/1000 residues), in which lysine and leucine were found at a high content as compared to other essential amino acids. Moreover, hydrophobic amino acids (33.04% of total amino acids), especially glycine and alanine, were the major amino acids. Glutamic acid (177.68 residues/1000 residues), an acidic amino acid, was also found in the A-0.2 sample.

### 3.8. Effects of the Digest Obtained from GIT Digestion at Various Concentrations on Caco-2 Cell Viability and Bioavailability

#### 3.8.1. Caco-2 Cell Viability

**Figure 5:**
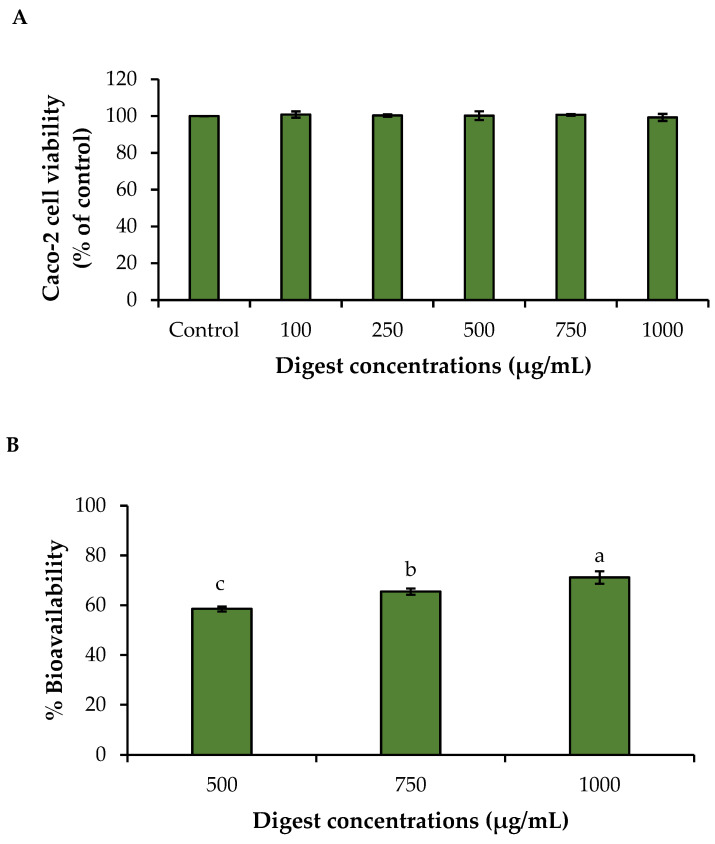
The impact of the A-0.2 digest obtained from GIT digestion at various concentrations on Caco-2 cell viability (**A**), and %Bioavailability of the digest from GIT digestion at the selected concentrations (**B**). Values are expressed as the mean ± SD (*n* = 3). Different lowercase letters (a–c) on the bar indicate significant differences (*p* < 0.05).

As illustrated in Figure 5A, the digest at all concentrations tested had no cytotoxicity, compared to the control (without the digest treatment) (*p* > 0.05) as indicated by cell viability ranging from 99.30% to 100.86%. The result revealed that the digest of A-0.2 had high biocompatibility to Caco-2 cells. Since the high doses (>250 μg/mL) and low doses (<250 μg/mL) did not alter cell viability, the former was selected for the bioavailability test via the Caco-2 monolayer.

#### 3.8.2. Bioavailability Assessment

After absorption and transportation via Caco-2 cells, α-amino group content (AAGC) was assessed. The percentage of bioavailability of the digest obtained from the A-0.2 sample ranged from 58.55% to 71.15% (Figure 5B). The increase in bioavailability was observed with augmenting concentrations of the digest (*p* < 0.05), indicating that peptides of the A-0.2 sample could permeate across the Caco-2 cell monolayer according to the concentration gradient.

## 4. Discussion

In the present study, ethanol was used to remove lipids from cricket powder. Different extraction times resulted in varying efficacies of lipid removal (Table 1). The highest efficacy of lipid removal was found when the extraction time using ethanol was 2 h (E-2) (*p* < 0.05). Ethanol is commonly used as the organic solvent for the defatting of cricket powder [13]. Prolonged extraction times along with agitation possibly promoted efficacy of lipid removal by enhancing the diffusion of the solvent into powder. Subsequently, lipids distributed in powder were more leached out. As a result, lowered lipid content was obtained. With increasing extraction time above 2 h, the equilibrium diffusion might be reached [25] and the migration of the solvent into powder becomes constant. Thus, the use of ethanol extraction for 2 h was an effective means for lipid removal of cricket powder.

When VI combined with ethanol was used (Table 2), increasing the VI cycle enhanced the efficacy of the defatting process of cricket powder (*p* < 0.05), in which diffusion of the solvent into powder might increase via deformation–relaxation and the hydrodynamic phenomenon. The internal air and liquid were more likely removed from the pores of powder and resulted in the increased gap available for the ethanol solution [14]. Thus, lipid extraction was increased with augmenting the VI cycle. Moreover, the efficacy of lipid removal in cricket powder treated with ethanol using four cycles of VI was greater than that of powder treated with ethanol alone (*p* < 0.05) (Table 2). The result supported that the defatting of cricket powder using ethanol with the aid of VI had more potential than using a solvent alone as indicated by higher lipid reduction (*p* < 0.05), compared to that defatted with the E-2 process (Table 2). Thus, the use of ethanol together with VI, especially at four cycles, could be implemented for the defatting of cricket powder.

When considering fatty acid profiles (Figure 2), a high amount of lipids was found in non-defatted cricket powder (control), particularly SFA, MUFA, and PUFA. High degrees of MUFA and PUFA are related to lipid oxidation, leading to the alteration of sensorial properties and shortened shelf-life of food products [26]. With defatting processes, cricket powder defatted with E-2 and VI-E-2 processes had a reduction in PUFA content, compared to the control, suggesting that the processes used could reduce lipids in cricket powder effectively. Nevertheless, higher lipid reduction was detected in the sample obtained from the VI-E-2 process as compared to that obtained from the E-2 process, in which the remaining MUFA and PUFA contents of the VI-E-2 sample were 23.84% and 35.70%, respectively. Lowering lipid contents mostly resulted from the use of the VI process, which could increase the efficacy of lipid removal from pores of powder. The result indicated that ethanol along with VI was a potential method for increasing the efficacy of the defatting of cricket powder via hydrodynamic and deformation–relaxation mechanisms as evidenced by lower residual MUFA and PUFA contents in the VI-E-2 sample.

Apart from those aforementioned results, lipid distribution using CLSM was also determined to monitor the amount of lipids distributed in the samples tested (Figure 3). The finding also confirmed that the VI-E-2 process could reduce lipid contents in cricket powder, which was similar to the results found in Table 2. This was governed by the aid of VI in increasing the migration of the solvent into the powder, thus lowering lipid content, especially MUFA and PUFA. Since the VI-E-2 sample showed the highest lipid reduction, this sample was selected for the further preparation of the protein hydrolysate using various enzymatic hydrolysis processes.

As shown in Table 3, defatted cricket protein hydrolysates (DC-PHs) prepared by Alcalase hydrolysis had a higher yield than those prepared by Flavourzyme hydrolysis at all the concentrations tested (*p* < 0.05). Higher yield likely resulted from Alcalase, which cleaved peptide bonds of protein in cricket powder during hydrolysis to a higher extent than Flavourzyme hydrolysis, leading to the increased yield of the obtained DC-PH, especially when the concentration of the enzyme increased up to 0.3 units/g dry sample (*p* < 0.05). Similarly, Muzaifa et al. [27] found that a protein hydrolysate from fish by-products digested by Alcalase provided higher protein content than that digested by Flavourzyme (*p* < 0.05). Also, the yield of the A-0.3 sample (21.56%) in the current study was higher than that of the protein hydrolysate from *Hermetia illucens* prepared by bromelain hydrolysis (10.7%) [28]. Thus, the type and level of the enzyme used play a crucial role in determining the yield of the protein hydrolysate.

When the α-amino group content was examined (Table 3), a similar result was observed to yield, in which Alcalase hydrolysis provided greater α-amino group content than Flavourzyme hydrolysis at the same enzyme levels tested (*p* < 0.05). It was demonstrated that hydrolysis with Alcalase could augment the cleavage of peptides in DC protein. In general, Alcalase has broad specificity, which preferably cleaves peptide bonds of hydrophobic amino acids [29], while Flavourzyme hydrolyzes the N-terminal of peptide bonds of aromatic amino acids. DC-PH more likely contained peptides with hydrophobic amino acids, resulting in the increased degree of hydrolysis when Alcalase was applied. Moreover, there was no difference in the α-amino group content for DC-PH prepared by Alcalase (A) at all the levels tested (*p* > 0.05), while the α-amino group content was decreased with increasing levels of Flavourzyme (F) (*p* < 0.05). Nonetheless, no difference in the α-amino group content between F-0.3 and F-0.4 samples was detected (*p* > 0.05). Therefore, the increased level of enzymes did not promote the degree of hydrolysis. The enzymatic reaction rate possibly became steady with enhancing levels of Alcalase, resulting in no change in α-amino group content [21]. Furthermore, excessive levels of the enzyme also resulted in the decrease in α-amino group content due to enzyme/substrate inhibition and inaccessibility of cleavage sites [30,31]. Thus, the level of the enzyme used was an important parameter affecting the degree of hydrolysis of DC-PH.

We considered antioxidant activities of DC-PH (Table 3). All samples tested could donate protons to radicals in both hydrophobic and hydrophilic systems as indicated by DPPH and ABTS radical-scavenging activities [31]. Also, they could reduce the TPTZ-Fe (III) complex to form the TPTZ-Fe (II) complex and chelated metal ions. According to the results in Table 3, antioxidant activities of DC-PH were in line with the α-amino group content, in which the highest ABTS radical-scavenging activity, FRAP, and MCA were found in the A-0.2 sample, compared to other samples (*p* < 0.05). It was revealed that Alcalase hydrolysis could produce higher antioxidative peptides than Flavourzyme hydrolysis. Also, the A-0.2 sample had higher MCA than ascorbic acid (*p* < 0.05), indicating that the protein hydrolysate prepared using Alcalase hydrolysis at 0.2 units/g dry sample could be an excellent antioxidant, especially as a metal chelator. Recently, Yeerong et al. [32] documented that a protein hydrolysate from house cricket prepared by Alcalase hydrolysis possessed high antioxidant activity. Three antioxidative peptides found in the protein hydrolysate from house cricket included Ala-Val-Thr-Lys-Ala-Asp-Pro-Tyr-Thr-Asp-Gln, Thr-Val-Met-Glu-Leu-Asn-Asp-Leu-Val-Lys-Ala-Phe, and Val-Pro-Leu-Leu-Glu-Pro-Trp [32]. Those peptides more likely had the ability to scavenge radicals. Moreover, peptides rich in hydrophobic amino acids could provide protons or electrons to radicals. Coincidentally, a high amount of those amino acids (33.04%) was observed in DC-PH (Table 4), corresponding to antioxidant activity [33]. Also, peptides containing glutamic acid, lysine, histidine, and arginine were reported to act as metal chelators [21,33,34]. Thus, the DC-PH contained peptides with several antioxidant activities. Based on the results in Table 3, the A-0.2 sample showed the highest antioxidant activities; thus, it was chosen for measuring amino acid composition and size distribution.

The amino acid composition of the A-0.2 sample is presented in Table 4. Glutamic acid, aspartic acid, alanine, and arginine were observed at high contents in the sample. Yeerong et al. [32] reported a similar result, in which a protein hydrolysate from house cricket prepared by Alcalase hydrolysis had high levels of glutamic acid, aspartic acid, and alanine. Cricket (*Gryllodes sigillatus*) peptides derived from simulated gastrointestinal digestion had glutamic acid and aspartic acid as the predominant amino acids [35]. The presence of those amino acids in the A-0.2 sample was likely associated with antioxidant activities, especially activities towards free radicals and metal chelating [36]. Apart from the aforementioned amino acids, the A-0.2 sample had a high amount of hydrophobic amino acids, which had a powerful antioxidant activity [35,37].

Additionally, the size distribution of peptides in the A-0.2 sample was assessed (Figure 4). Molecular weight (MW) of A-0.2-derived peptides ranged from 493 Da to 7248 Da. Consistent with previous studies, peptides obtained from cricket prepared by Alcalase hydrolysis at 2.1% contained peptides having MW below 15 kDa [32]. Cricket (*Gryllus bimaculatus*) protein hydrolyzed by Alcalase at 3% had peptides with MW ranging from 10 to 75 kDa [9]. Different raw materials, processes, and levels of the enzyme used probably contributed to different MWs, in which biological activities of peptides were different [38]. Mostly, short-chain peptides provided greater antioxidant activity than long-chain peptides as documented by several studies [39,40,41]. The peptides from black cricket (*Gryllus assimilis*) with MW < 3 kDa exhibited high radical-scavenging activity [10]. Thus, DC-PH prepared by the A-0.2 process could be a promising natural antioxidant.

Apart from characterizations, the biocompatibility effect of the digest obtained from the A-0.2 sample on Caco-2 cells was also investigated, where cell viability was tested by the MTT assay as displayed in Figure 5A. There was no cytotoxicity after treatment with the digest at all concentrations (100–1000 µg/mL), indicating that the digest obtained from the A-0.2 sample had high biocompatibility toward cells. Since all concentrations could apply in cells without toxicity, high doses (500–1000 µg/mL) of the digest were selected for assessment of bioavailability via the Caco-2 monolayer.

As presented in Figure 5B, AAGC of the permeates was increased with enhancing the levels of the digest in a dose-dependent manner (*p* < 0.05). This implied that peptides of the digest were mostly absorbed through the cells depending on the concentrations used. In general, the transportation of peptides can occur through paracellular and transcellular pathways of Caco-2 cells [42]. With increasing levels of the digest, the amount of the peptides presumably permeated in the basolateral side in different ways such as PepT1, paracellular, and transcytosis routes [43]. Di/tri-peptides can be conveyed by the PepT1 route, particularly peptides with basic amino acids [44]. Peptides of the A-0.2 sample with basic amino acids are more likely linked to the binding site of PepT1, resulting in enhanced accessibility. Furthermore, oligopeptides can be transported via the paracellular tight-junction route [45]. Also, peptides rich in hydrophobic amino acids permeate cell membranes by interacting with the hydrophobic binding site of PepT1 [45]. Apart from PepT1-mediated transport, the paracellular pathway facilitates the transportation of negatively charged peptides with a small MW [46], while transcytosis prefers to transport positively charged peptides and hydrophobic peptides with large MW [46]. Thus, the permeability of the A-0.2 sample across the Caco-2 cell monolayer was possibly governed by the digest concentrations and transportation pathways.

## 5. Conclusions

The ethanol with the aid of the VI process for 2 h could be used as a process to enhance the efficacy of lipid removal of cricket powder, in which lipids were removed by 86.83%. This process could also reduce MUFA and PUFA contents in the sample. The use of Alcalase hydrolysis, especially at 0.2 units/g dry sample, could increase the degree of hydrolysis of peptides of the protein hydrolysate from defatted cricket powder (DC-PH) and provided the highest antioxidant activities as assayed by FRAP and ABTS radical-scavenging activity. The resulting protein hydrolysate contained high hydrophobic amino acids and peptides with various MWs. The digest from GIT was not only compatible to Caco-2 cells but could also absorb and transport via cells following the concentration gradient (500–1000 µg/mL). Thus, DC-PH could be a potent functional ingredient with safety. Further research is the application of DC-PH in food products and the study on their stability during storage. Also, the extracted oils will be further separated and purified to produce functional ingredients.

## Figures and Tables

**Table 1:** Impact of ethanol at various extraction times on defatting of cricket powder.

Defatting Time (h)	Lipid Removal (%)
1	78.42 ± 1.28 ^b^
2	80.38 ± 0.66 ^a^
3	81.15 ± 1.08 ^a^
4	81.73 ± 0.70 ^a^

Values are expressed as mean ± SD (*n* = 3). Different letters within same column indicate significant differences (*p* < 0.05).

**Table 2:** Impact of ethanol in combination with vacuum impregnation (VI) with various VI cycles on defatting of cricket powder.

VI Cycle	Lipid Removal (%)
1	81.96 ± 1.15 ^c,C^
2	82.86 ± 0.28 ^bc,BC^
3	83.86 ± 0.43 ^b,B^
4	86.83 ± 0.78 ^a,A^
E-2	80.38 ± 0.66 ^d^

Data are expressed as mean ± SD (*n* = 3). Different lowercase superscripts indicate significant differences among all samples tested (*p* < 0.05). Different uppercase superscripts indicate significant differences among VI cycles used (*p* < 0.05). VI process was performed at 10 ± 5 kPa vacuum pressure. One cycle of VI process took 15 min for vacuum time, followed by restoration to atmospheric pressure for 10 min with total operation time of 2 h. E-2: cricket powder defatted with ethanol for 2 h.

**Table 3:** Effect of different enzymatic hydrolysis processes on yield, **α**-amino group content, and antioxidant activities of protein hydrolysate from defatted cricket powder.

Samples	%Yield	α-Amino Group Content (mmol L-Leucine/g Dry Sample)	Antioxidant Activities			
			ABTS Radical-Scavenging Activity (µmol TE/g Dry Sample)	DPPH Radical Scavenging Activity (µmol TE/g Dry Sample)	FRAP (µmol TE/g Dry Sample)	MCA (µmol EDTA/g Dry Sample)
A-0.2	20.01 ± 0.11 ^b,B^	6.27 ± 0.05 ^a,A^	19.53 ± 0.15 ^a,B^	6.50 ± 0.10 ^a,C^	5.90 ± 0.10 ^a,B^	6.48 ± 0.33 ^a,A^
A-0.3	21.56 ± 0.11 ^a,A^	6.24 ± 0.13 ^a,A^	17.33 ± 0.82 ^b,C^	6.88 ± 0.10 ^a,B^	5.38 ± 0.14 ^b,C^	5.37 ± 0.31 ^b,C^
A-0.4	21.24 ± 0.36 ^a,A^	6.22 ± 0.05 ^a,A^	17.39 ± 0.25 ^b,C^	6.86 ± 0.12 ^a,B^	5.37 ± 0.07 ^b,C^	5.23 ± 0.13 ^b,C^
F-0.2	9.72 ± 0.17 ^b,D^	4.85 ± 0.05 ^a,B^	15.36 ± 0.19 ^a,D^	5.64 ± 0.13 ^a,D^	3.42 ± 0.07 ^a,D^	6.32 ± 0.07 ^a,A^
F-0.3	11.69 ± 0.09 ^a,C^	4.79 ± 0.01 ^ab,B^	15.19 ± 0.15 ^a,D^	5.58 ± 0.12 ^b,DE^	2.63 ± 0.07 ^b,E^	5.95 ± 0.05 ^b,B^
F-0.4	11.13 ± 0.82 ^a,C^	4.73 ± 0.05 ^b,B^	15.09 ± 0.15 ^a,D^	5.35 ± 0.22 ^b,E^	2.61 ± 0.02 ^b,E^	6.16 ± 0.05 ^b,B^
AA	-	-	21.88 ± 0.53 ^A^	764.00 ± 10.14 ^A^	530.67 ± 6.29 ^A^	5.04 ± 0.27 ^C^

Data are expressed as the mean ± SD (*n* = 3). Different uppercase superscripts within the same column indicate significant differences among all the samples tested (*p* < 0.05). Different lowercase superscripts within the same column under the same enzyme used indicate significant differences among the different enzyme concentrations used (*p* < 0.05). A-0.2 to A-0.4: protein hydrolysate from defatted cricket powder prepared using Alcalase at 0.2, 0.3, and 0.4 units/g dry sample, respectively. F-0.2 to F-0.4: protein hydrolysate from defatted cricket powder prepared using Flavourzyme at 0.2, 0.3, and 0.4 units/g dry sample, respectively. AA: ascorbic acid at 0.1 mg/mL. FRAP: ferric reducing antioxidant power; MCA: metal chelating activity.

**Table 4:** Amino acid compositions of A-0.2 sample.

Amino Acids	Residues/1000 Residues
Aspartic acid	96.16 ± 4.60
Cystine	2.22 ± 0.00
Glutamic acid	177.68 ± 5.14
Glycine	80.11 ± 4.94
Histidine	28.69 ± 0.02
Hydroxylysine	0.47 ± 0.07
Hydroxyproline	1.40 ± 0.04
Isoleucine	25.74 ± 0.03
L-Alanine	97.25 ± 4.67
L-Arginine	126.51 ± 0.94
Leucine	54.74 ± 5.44
Lysine	65.36 ± 0.23
Methionine	9.61 ± 0.04
Phenylalanine	19.72 ± 0.11
Proline	71.38 ± 4.96
Serine	42.91 ± 4.79
Threonine	33.24 ± 0.48
Tryptophan	4.13 ± 0.04
Tyrosine	23.61 ± 0.05
Valine	39.04 ± 4.60
Total amino acids	1000.00
Hydrophobic amino acids	330.36 ± 19.87
Essential amino acids	387.07 ± 11.82

A-0.2: protein hydrolysate from defatted cricket powder prepared using Alcalase at 0.2 units/g dry sample. Data were analyzed in duplicate.

## Data Availability

The original contributions presented in the study are included in the article; further inquiries can be directed to the corresponding author.
